# Vision-based Pakistani sign language recognition using bag-of-words and support vector machines

**DOI:** 10.1038/s41598-022-15864-6

**Published:** 2022-12-09

**Authors:** Muhammad Shaheer Mirza, Sheikh Muhammad Munaf, Fahad Azim, Shahid Ali, Saad Jawaid Khan

**Affiliations:** 1 Department of Biomedical Engineering, Faculty of Engineering, Science, Technology and Management, Ziauddin University, Karachi, Pakistan; 2 Department of Software Engineering, Faculty of Engineering, Science, Technology and Management, Ziauddin University, Karachi, Pakistan; 3 Department of Electrical Engineering, Faculty of Engineering, Science, Technology and Management, Ziauddin University, Karachi, Pakistan; 4 Department of Speech Language and Hearing Sciences, Faculty of Health Sciences, Ziauddin University, Karachi, Pakistan

**Keywords:** Engineering, Mathematics and computing

## Abstract

In order to perform their daily activities, a person is required to communicating with others. This can be a major obstacle for the deaf population of the world, who communicate using sign languages (SL). Pakistani Sign Language (PSL) is used by more than 250,000 deaf Pakistanis. Developing a SL recognition system would greatly facilitate these people. This study aimed to collect data of static and dynamic PSL alphabets and to develop a vision-based system for their recognition using Bag-of-Words (BoW) and Support Vector Machine (SVM) techniques. A total of 5120 images for 36 static PSL alphabet signs and 353 videos with 45,224 frames for 3 dynamic PSL alphabet signs were collected from 10 native signers of PSL. The developed system used the collected data as input, resized the data to various scales and converted the RGB images into grayscale. The resized grayscale images were segmented using Thresholding technique and features were extracted using Speeded Up Robust Feature (SURF). The obtained SURF descriptors were clustered using K-means clustering. A BoW was obtained by computing the Euclidean distance between the SURF descriptors and the clustered data. The codebooks were divided into training and testing using fivefold cross validation. The highest overall classification accuracy for static PSL signs was 97.80% at 750 × 750 image dimensions and 500 Bags. For dynamic PSL signs a 96.53% accuracy was obtained at 480 × 270 video resolution and 200 Bags.

## Introduction

In today’s fast-growing world, communication is key, whether it is communication between different machines, between people or both of them combined. A person cannot perform their everyday tasks without communicating with others. This poses a major problem for the deaf population of the world. According to the World Health Organization, around 466 million people worldwide have disabling hearing loss, which are estimated to increase to over 900 million people by 2050^1^.

The deaf people rely on sign languages (SL), native to their countries, to communicate with others and this is an issue that still remains because not all people are familiar with their local sign languages. Researchers around the world have been working to bridge this communication gap between the deaf and the normal population and have come up with a solution, i.e., automated sign language recognition systems.

According to the Pakistan Association of the Deaf, there are approximately 250,000 hearing-impaired Pakistanis^2^, and many of them use Pakistani Sign Language (PSL) as a medium of communication. Developing a SL recognition system would be greatly beneficial for these people. In all the studies mentioned in the next section, only a few have used PSL in their SL recognition systems which means that vision-based Pakistani SL recognition is still a relatively unexplored area of research.

The studies mentioned in the literature review, give us the overall layout of all the techniques used for various SL recognition systems. These techniques can be explored for developing PSL recognition systems. Vision-based PSL alphabets’ datasets consisting of bare-handed images and videos, i.e., without any sensors, are not publicly available so researchers have to collect their own dataset in order to perform their studies. The datasets that are available either use sensors to detect PSL signs or are of PSL words. The proposed system will use image for static (still) signs and videos for dynamic (signs that require movement of the hand) signs of PSL alphabets. All previous PSL studies only focused on static PSL alphabets and none have used dynamic PSL alphabets and only dynamic PSL words have previously been classified. Feature extraction techniques such as SURF, have not yielded good accuracies while being used with SVM and Bag-of-Words (BoW) technique has yet to be applied on vision-based PSL recognition systems.

Therefore, a vision-based PSL alphabets recognition system will be developed in this study, that will form BoW using SURF features and K-means clustering and classify the obtained codebooks of static and dynamic PSL alphabets using Support Vector Machines.

The objectives of this research are as following:

1. To create a dataset containing static and dynamic PSL alphabets, with uniform background and lighting conditions.
2. To develop a vision-based system for the recognition of Pakistani Sign Language (PSL) alphabets using Bag-of-Words (BoW) and Support Vector Machine (SVM) techniques.

The paper is organized as follows: second section explains the methods used for the literature review and the related studies obtained; third section describes the approach in this study, including the data collection protocol used, and the techniques used for the recognition of PSL alphabets; fourth section provides the experimental results and their discussion; fifth section concludes this paper.

## Literature review

Several studies have been performed to develop SL recognition systems using different image processing and learning methods. Most of these studies extract specific features and then use machine learning algorithms to classify the SL images. Many different SL have been used in these studies, namely American^3–11^, Amharic SL^12^, Arabic SL^13–17^, British SL^18,19^, Chinese SL^20,21^, German SL^22,23^, Indian SL^24^, Mexican SL^25^, Pakistani SL^26–31^, Persian SL^32^, and more in combination such as American and German SL^33^, American and Thai SL^34^ and American and Indian SL^35^.

The literature review done of the SL mentioned, was focused between the time period of 2010 and 2021. Instead of sensor-based recognition systems, i.e., systems that use Cyber-gloves, leap motion controller, accelerometers or EMG sensors, vision-based SL recognition systems were focused. Specifically, those systems that used images and videos from a single camera of bare hands, instead of those that used multiple cameras or different object tracking technologies for their study. Many systems used a combination of image and video-based datasets as input and used different classifiers, such as, Neural Networks like Convolutional Neural Network (CNN) and Multilayer Perceptron (MLP), Support Vector Machine (SVM), K Nearest Neighbor (KNN), Hidden Markov Model (HMM), etc. to recognize their respective SLs.

Singha et al., used dynamic American SL and features including location, position, velocity, acceleration, orientation, distance and many more to obtain an accuracy of 92.23% using a fusion of classifiers like KNN, SVM and Artificial NN^5^. Dardas et al., used the Bag-of-features technique with Scale Invariant Feature Transform (SIFT) and SVM to achieve 96.23% accuracy of static American SL^8^. Inception v3 CNN with SVM was used by Abiyev et al., to obtain a 99.90% accuracy for classification of American SL^10^. AlexNet and VGG16 were used with SVM by Barbhuiya et al., to classify static American SL to obtain 99.82% and 99.76% accuracies, respectively^11^. Tamiru et al., collected Amharic SL and extracted shape features using Fourier descriptor (FD), motion features such as direction and angle and colour feature to obtain a 98.06% accuracy using SVM^12^. Dahmani et al., extracted Tchebichef moments, Hu moments and geometric features from Arabic SL to classified them using SVM to obtain a 96.88% accuracy^17^. Charles et al., used dynamic British SL signs from TV broadcasts used Histogram of gradients with K-means clustering and SVM to obtain a classification accuracy of 75%^19^.

Cheng et al., collected static Chinese SL and extracted features from palm centroids, their key points, and the Euclidean distance between them and, performed feature reduction using uncorrelated linear discriminant analysis (ULDA). Then Dynamic Time Warping (DTW)-distance-based feature mapping was used in combination with SVM to obtain a 99.03% accuracy^21^. Athira et al., used Indian SL with Zernike moments and centroid of signs to recognize static signs with 90.1% and dynamic signs with 89% accuracies using SVM^24^. Cabrera et al., obtained a 96.27% accuracy by classifying dynamic Mexican SL using SVM and Geometric features, such as Fourier descriptors, Hu moments, Ellipse, Gupta descriptors and Flusser moments^25^. Joshi et al., used static American and Indian SL, using shape-based features and using SVM obtained accuracies of 98.6% using Indian SL with uniform background, and 98.8% using Jochen–Triesch static hand posture with uniform background datasets^35^.

The literature review was done for Pakistani SL (PSL) to identify the protocols used for the collection of data for static and dynamic PSL alphabets and the methods used for the recognition of PSL alphabets. The protocol used by the researchers of all the included PSL studies used RGB images and single-handed static signs of PSL alphabets except for Saqib et al., who used dynamic PSL words^31^. The studies used various lighting conditions and studies by Kausar et al.^26^, and Shah et al.^30^, mentioned that the clothing should be separate from the skin colour of the participant. Khan et al.^29^, and Ahmed et al.^28^, used complex backgrounds to collect the data while the rest used uniform backgrounds.

Khan et al., collected a total of 500 (426 training/74 testing) images of 37 PSL alphabets, converted the RGB images to grayscale, segmented based on skin colour, resized the images to 300 × 400 pixels, applied Discrete Wavelet Transform (DWT) to extract features and achieved 84.6% classification accuracy using MLP^29^. Ahmed et al., used 10 PSL alphabets and collected 600 (360 training/240 testing) images from 60 participants, resized them to 640 × 480, used ROI segmentation in HSV color space to extract skin pixels, extracted global features including length, area, rectangularity, eccentricity, and more and shape features and used multi-class SVM to obtain an 83% accuracy^28^. 80% accuracy was obtained by Kausar et al., using 37 Urdu alphabets and 9 numbers, totaling to 455 images (245 training/210 testing), K-means clustering based segmentation, centroid distance signature in mathematical modelling (polynomial, sinusoidal, exponential, gaussian) and KNN^26^. Multiclass SVM was used by Shah et al., to achieve 77.18% accuracy, with six statistical features of local binary pattern histogram i.e., standard deviation, variance, skewness, kurtosis, entropy and energy, with skin detection being done in HSV domain from 3414 images (2384 training/1030 testing), using 37 PSL alphabets^27^.

Saqib et al., used 20 dynamic PSL words, with 8000 videos (6480 training/1520 testing) collected from 15 participants, resized the images to 234 × 234 and converted them to grayscale, and used CNN with Convolution layers and fully connected layers, along functional layers such as max pooling Layers, Rectified Linear Units layer (ReLU layer) and SoftMax activation function to achieve a 90.79% accuracy^31^. Shah et al., classified 36 PSL alphabets, with 6633 images (4643 training/1990 testing) collected from 6 participants using SVM and using K-means clustering-based segmentation and converting them to grayscale, obtained classification accuracies of 15.41% using Speeded Up Robust Features (SURF), 87.67% using Edge Orientation Histogram (EOH), 45.71% using Local Binary Patterns (LBP), and 89.52% using Histogram of Oriented Gradient (HOG) and the final reported accuracy of 91.98%^30^.

## Methodology

The methodology for this study was divided into 2 parts:

- Data Collection and,
- Data Analysis.

### Data collection

**Figure 1:**
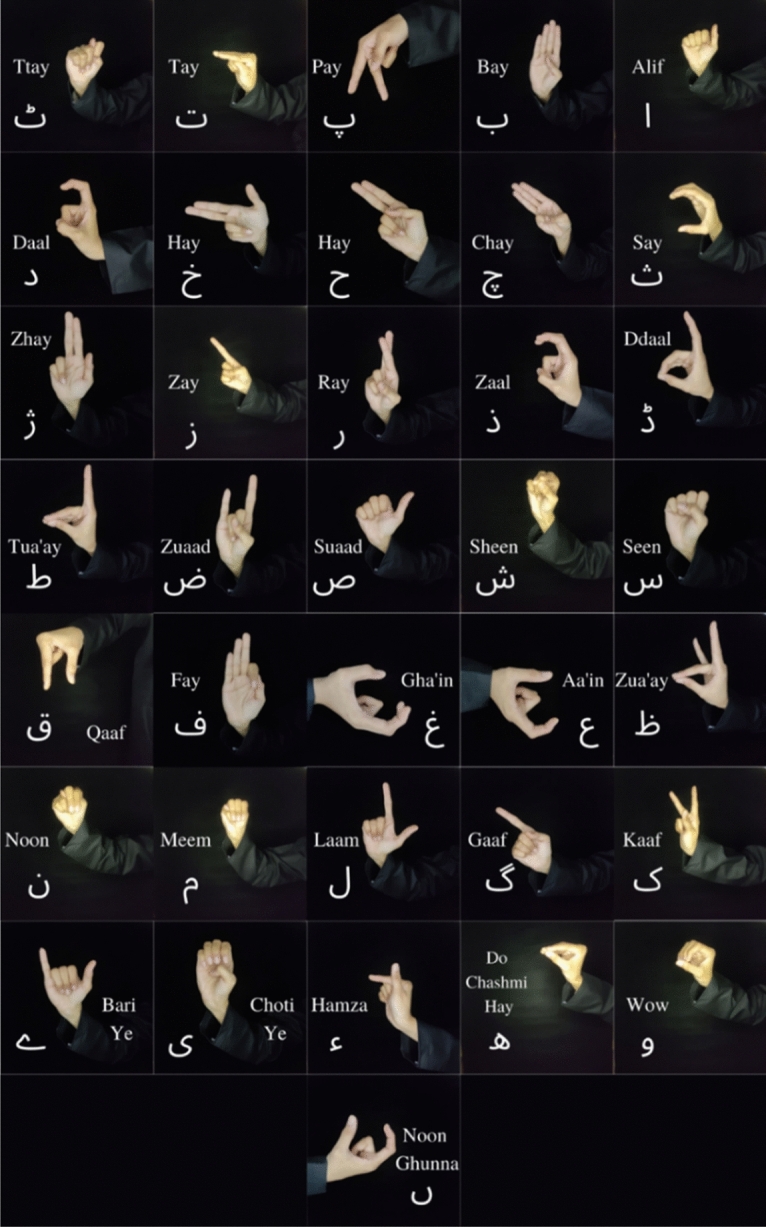
PSL static alphabets.

**Figure 2:**
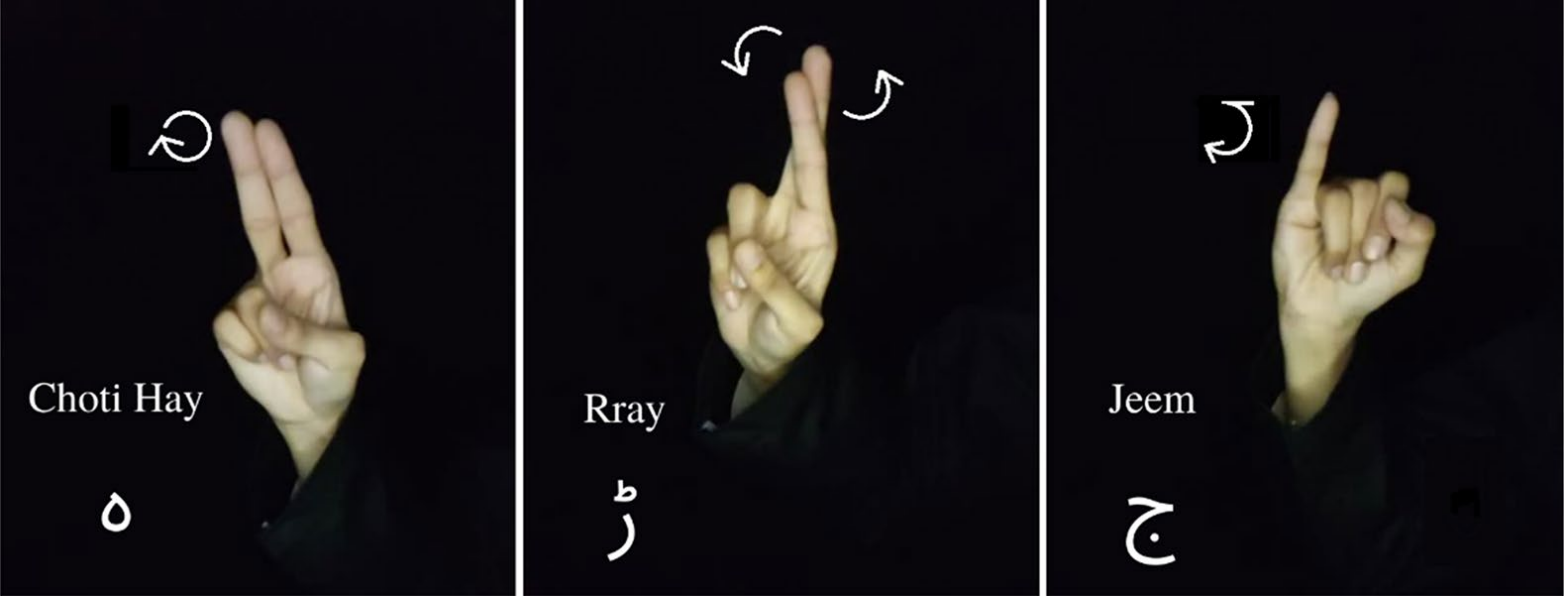
PSL dynamic alphabets.

The data was collected for this study over the course of three months at Ziauddin College of Speech Language and Hearing Sciences, Ziauddin University, Clifton, Karachi. The data collection protocols were approved by the Ziauddin University Ethical Review Committee (Reference Code: 4611221SJBME) and the data collected was in accordance to their guidelines and regulations. Native signers of PSL were selected as participants for this study, irrespective of their race, gender, age, height and skin colour and their written informed consent was obtained. The protocol used for the collected data is mentioned in Table 1. A total of 39 signs of PSL alphabets were collected for this study, i.e., 36 static signs and 3 dynamic signs, as specified in the Figs. 1 and 2, respectively.

**Table 1:** PSL data collection protocol.

Parameters	Our dataset
Imaging technique used	48MP smart phone camera
Image dimensions	3000 × 3000
Video resolution and frames per second	1920 × 1080 (1080p) at 60fps
image and video type	RGB
Hands used in performing signs	One Hand
Static signs	Images of the hand
Dynamic signs	Videos of the signer
Clothing requirements	Uniform clothing for all the participants
Lighting conditions	Uniform lighting
Background conditions	Uniform background
Total number of signs	36 Static Urdu alphabets + 3 Dynamic Urdu alphabets
Number of images/videos	At least 10 samples per sign per participant
Number of participants	10
Selection of participants	Native PSL users who can perform the required signs

The participants were provided with a black lab coat to keep the same clothing conditions and asked to stand in front of the camera with black background. A separate white light source was attached with the camera with uniform intensity for all the participants. The height and the distance between the camera and the participant were not constant. The participants were then asked to perform the signs as they naturally would and the images and videos were captured.

### Data analysis

**Figure 3:**
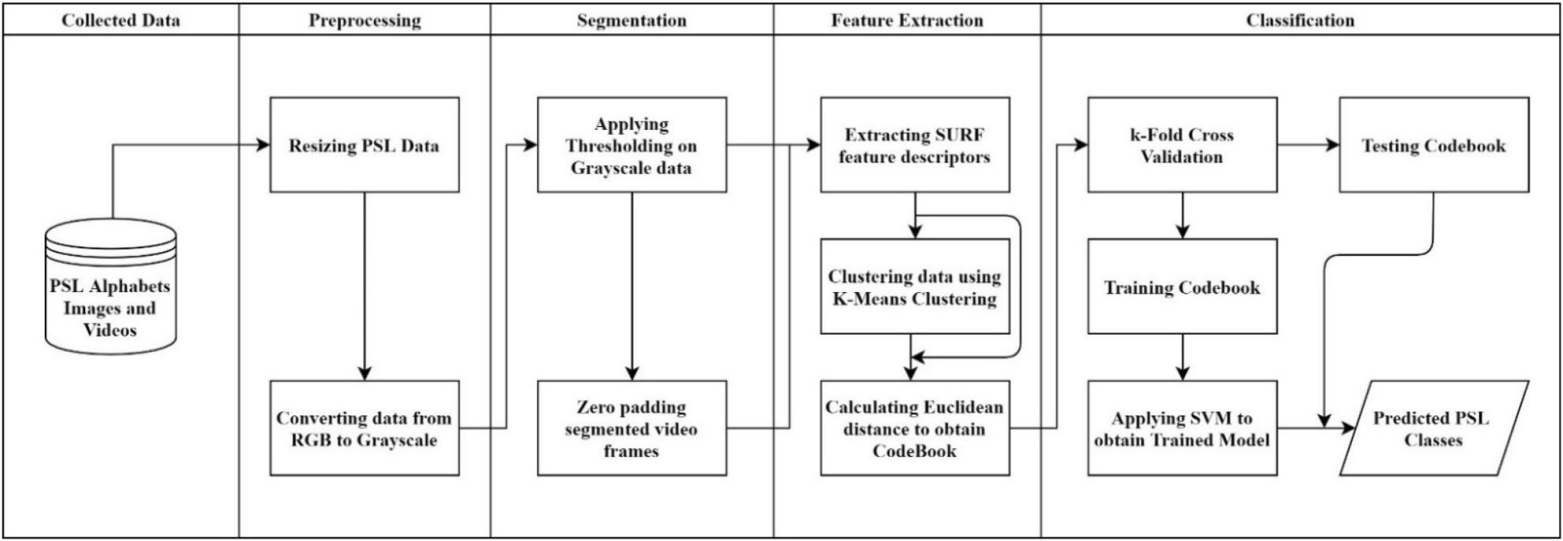
PSL recognition flowchart.

The images and videos from the collected data were stored in labelled folders. The videos were processed frame by frame, act as static images. The flowchart for the entire data analysis processing is shown in Fig. 3.

#### Preprocessing

The collected data was resized to different scales of the original images and videos, i.e., 0.125 (375 × 375), 0.25 (750 × 750), 0.375 (1225 × 1225) and 0.5 (1500 × 1500) for images and 0.125 (240 × 135), 0.25 (480 × 270) and 0.375 (720 × 405) for videos. Once the images were resized, they were converted from RGB to grayscale, in order to reduce their complexity and computation time.

#### Segmentation

The hand sign was detected by applying a threshold on the grayscale images whose value was set low enough to capture all the skin components in that image. As the grayscale pixel value ranges from 0 to 255, an initial threshold value was randomly selected and applied on all the PSL data. These values were then manually adjusted by checking the data before and after segmentation. The final threshold value was manually set at 105 for static and 100 for dynamic signs and applied on all the hand signs’ data. The black background and the black clothing conditions facilitated this process of thresholding.

**Figure 4:**
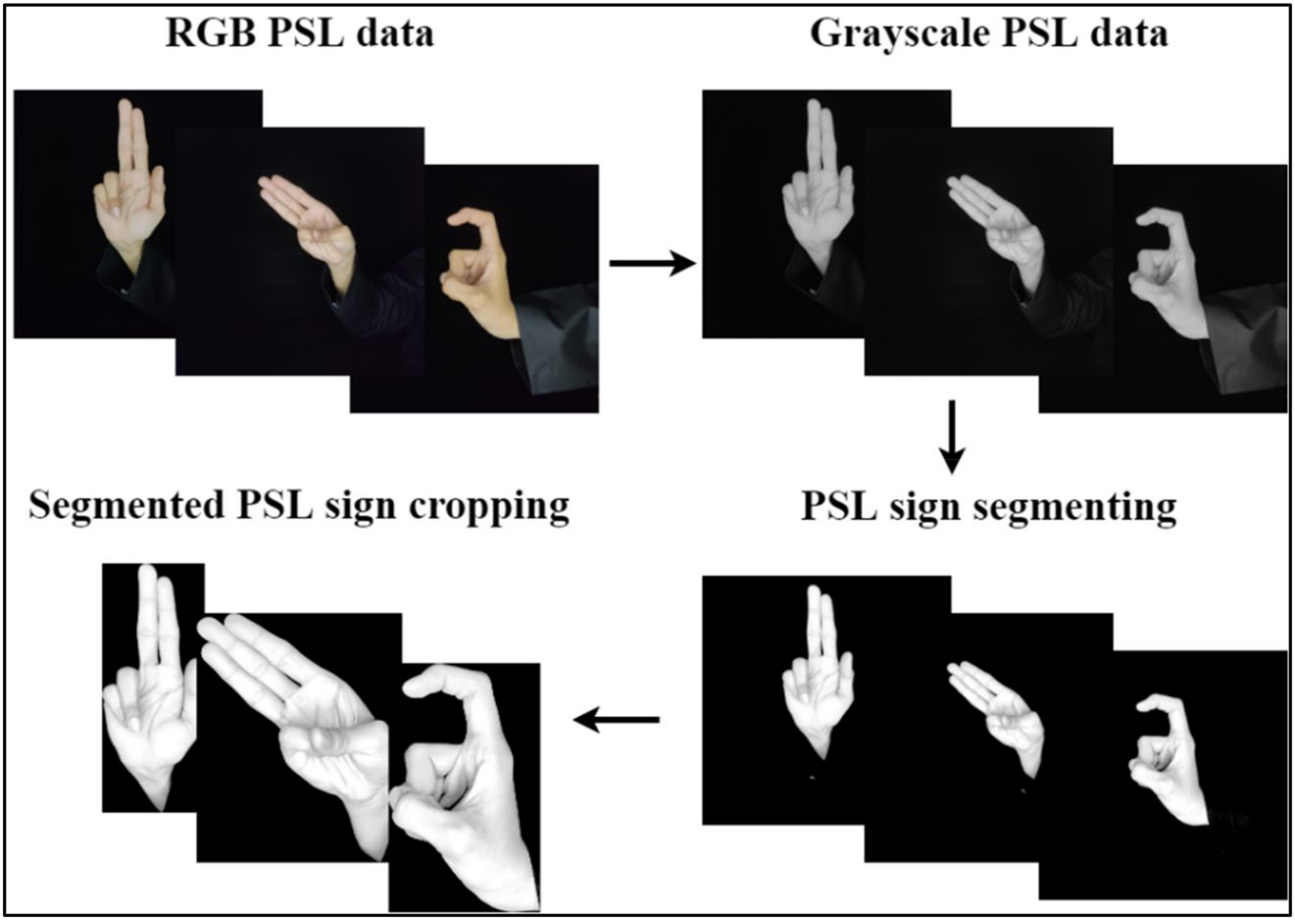
PSL data segmentation.

To crop the segmented hand sign, the bounding box technique was used. The thresholded signs were bound in boxes and their areas were calculated. A single image contained multiple skin components including the hand signs. The bounded box that had the largest area in the image, i.e., the hand sign, was cropped from each image and saved as the segmented image. The remaining skin components were excluded from the final segmented data. The segmented images obtained were of different dimensions, according to the signs being performed in the images. For videos, a uniform resolution size was required for segmented frames of a specific sign in order to save the cropped video. Zero padding was applied to convert all the segmented frames into uniform resolution. This process is shown in Fig. 4 and further discussed in Sect. 4 of this study.

#### Feature extraction

The SURF algorithm was applied on the images to extract their SURF features. The SURF points were detected for each image and then these points were used to extract the key point descriptors which are also called the SURF features. The same method was used for dynamic sign videos. As videos are a series of images or frames, each frame of every video was considered as an image and their features were extracted.

The SURF algorithm is based on the Hessian matrix^36^, because of its better performance in the required computation time and the overall detection accuracy. It relies on the determinant of Hessian for the selection of both, the scale and the location. Given a point $x=(x,y)$ in an image *I,* the Hessian matrix $H(x,\sigma )$ in $x$ at scale $\sigma$ is defined as follows

1 $$H\left(x,\sigma \right)= \left[\begin{array}{cc}{L}_{xx}\left(x,\sigma \right)& {L}_{xy}\left(x,\sigma \right)\\ {L}_{xy}\left(x,\sigma \right)& {L}_{yy}\left(x,\sigma \right)\end{array}\right]$$

where ${L}_{xx}\left(x,\sigma \right)$ is the convolution of Gaussian second order derivative $\frac{{\partial }^{2}}{{\partial x}^{2}}g(\sigma )$ with the image *I* in point $x$, and similarly for ${L}_{xy}\left(x,\sigma \right)$ and ${L}_{yy}\left(x,\sigma \right)$.

The key point descriptors in SURF were detected by first, constructing a circular region around the key points and then computing the Haar-wavelet responses in both x and y directions to get the orientation. Then using this orientation, a square region was constructed around the interest points. The square regions were split into 4 × 4 sub regions, to contain the relevant spatial information. Haar-wavelet responses ${d}_{x}$ and ${d}_{y}$ were weighted with a Gaussian centered at the interest point and summed over each sub region. The sum of the absolute values of the responses were also calculated $\left|{d}_{x}\right|$ and $\left|{d}_{y}\right|$, to extract information about the polarity of intensity changes. With this, each sub region had a four-dimensional descriptor vector,

2 $$v=\left(\sum {d}_{x},\sum {d}_{y},\sum \left|{d}_{x}\right|,\sum \left|{d}_{y}\right|\right)$$

This produced the standard SURF descriptor of length 64 for all 4 × 4 sub regions.

These extracted features of all the images were then clustering using unsupervised learning algorithm, K-means ++ clustering. The k-means ++ algorithm uses a heuristic method to find centroid seeds^37^.

The algorithm chooses seeds as follows, assuming the number of clusters is $k$. It then selects a descriptor at random from the images features dataset, $X$. The chosen descriptor is the first centroid, and is denoted ${c}_{1}$. It then computes the distances from each descriptor to ${c}_{1}$. The distance between ${c}_{j}$ and the descriptor $k$ as is denoted as $d({x}_{m},{c}_{j})$. Then it selects the next centroid, ${c}_{2}$ at random from $X$ with probability

3 $$\frac{{d}^{2}({x}_{m},{c}_{1})}{\sum_{j=1}^{n}{d}^{2}({x}_{j},{c}_{1})}$$

In order to choose center $j$, it computes the distances from each descriptor to each centroid, and assign each descriptor to its closest centroid. For $m=1,\dots ,n$ and $p=1,\dots ,j-1$, it selects the centroid $j$ at random from $X$ with probability

4 $$\frac{{d}^{2}({x}_{m},{c}_{p})}{\sum_{\{h;{x}_{h}\in {C}_{p}\}}{d}^{2}({x}_{h},{c}_{h})}$$

where ${C}_{p}$ is the set of all descriptor closest to centroid ${c}_{p}$ and ${x}_{m}$ belongs to ${C}_{p}$, i.e., it selects each subsequent center with a probability proportional to the distance from itself to the closest center that was already chosen. The process to choose the center $j$, is repeated until $k$ centroids are chosen.

A set of K-cluster values were used to form Bags (clusters) for the extracted features and each Bag is called a visual word. A set of these Bags form the visual vocabulary which are in-turn used to form the codebook or Bag-of-words. To select the K-cluster values for Bag formation, the maximum number of SURF descriptors were found for each scale of images and videos used, which were 90, 202, 307 and 444 for 375 × 375 (0.125), 750 × 750 (0.250), 1225 × 1225 (0.375) and 1500 × 1500 (0.500), image dimensions (scale), respectively, for static signs and 84 for all video resolutions (scale) used i.e., 240 × 135 (0.125), 480 × 270 (0.250), 720 × 405 (0.375) for dynamic signs. Using these maximum descriptors, 500 K-cluster value (Bag) was selected for static signs and 200 K-cluster value (Bag) was selected for dynamic signs.

An empty codebook was used to start the process. The Euclidean distance between each surf descriptor or feature and the centroid for each Bag and the feature was calculated. The least value of Euclidean distance was then assigned to the codebook as a part of that Bag using the formula,

5 $$d\left({x}_{i},{c}_{i}\right)=\sqrt{\sum {\left({x}_{i}-{c}_{i}\right)}^{2}}$$

where $d\left({x}_{i},{c}_{i}\right)$ is the distance between and the descriptor ${x}_{i}$ and the centroids ${c}_{i}$.

**Figure 5:**
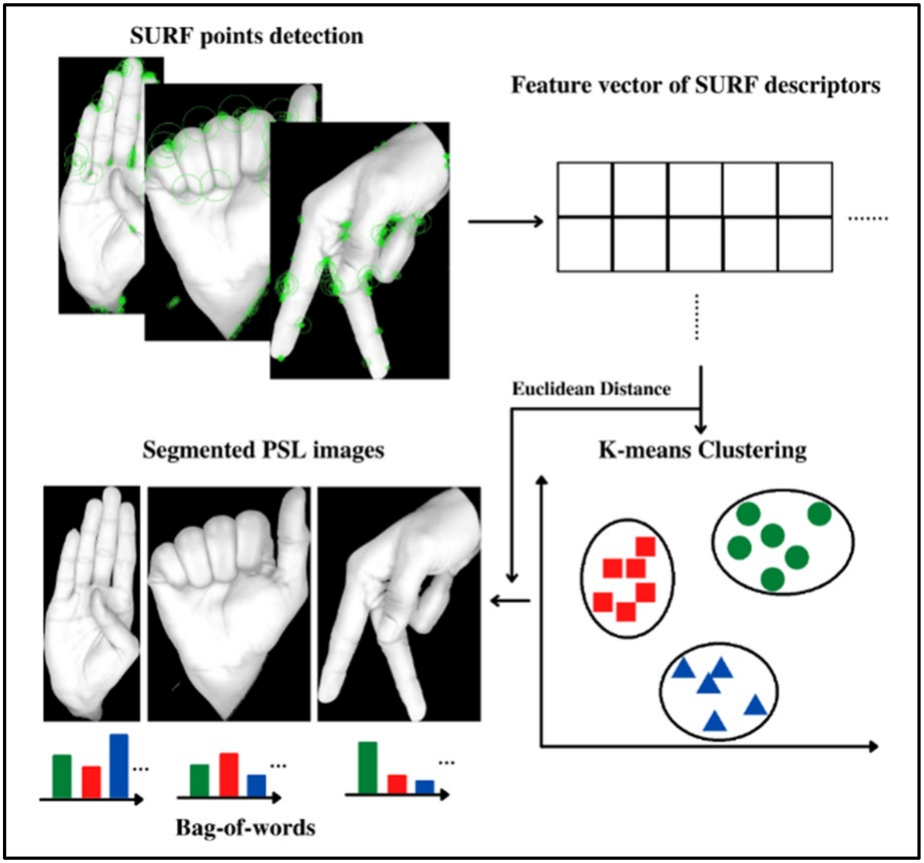
Bag-of-words generation.

The same procedure was repeated until each and every feature of all the images was assigned a Bag. If a specific Bag matched with more than one descriptor, the number of descriptors were added up. The final codebook obtained contained the number of features that each centroid had the least distance with, or the number of times each centroid was activated. The codebook obtained had the dimensions of the K-cluster value used and the total number of images. The labels for each image were then added to the codebook. This process of generating the codebook is shown in Fig. 5. The obtained codebook was then used for the classification of these images.

#### Classification

In k-fold cross-validation, the dataset being was partitioned into k disjoint subsets, known as folds, of approximately equal size. This partitioning is randomly performed by sampling the dataset without replacement.

**Figure 6:**
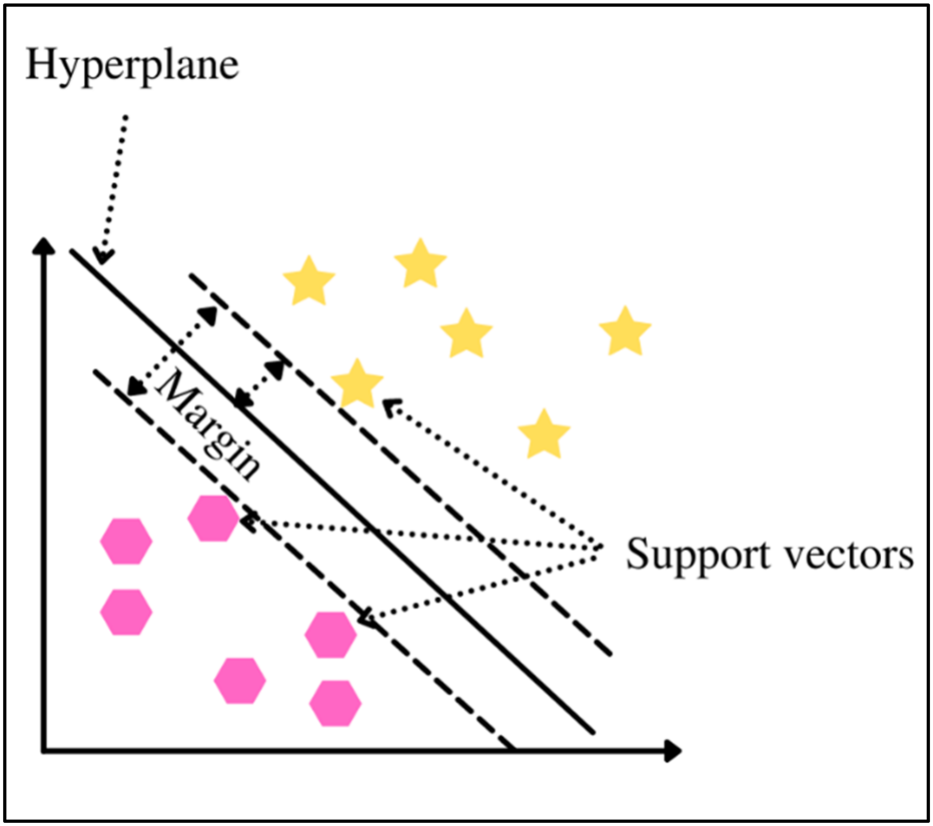
Margin Optimization.

The Support Vector Machine classifier (SVM) was used for classification. SVM used a part of the partitioned dataset, the training set, to find the optimal separating hyperplane between classes of the training data. The feature vectors near the hyperplane, the support vectors, are shown in Fig. 6. The SVM classifier used the training dataset to build a model that predicted whether the given example fell into one class of the target variable or the other.

The value of $k=5$ was chosen for k-fold cross-validation in this study, which partitioned the combined dataset, containing all the participants’ data according to their classes, into 80% for training and 20% for testing. As the dataset was folded five times, five training and five testing datasets were obtained, and the five training datasets were used to train five SVM models.

The validation or testing dataset was applied on the trained models, and the performance was measured. This process was repeated until all of the $k$ subsets served as testing sets. The cross-validated accuracy was obtained, by averaging the five accuracies achieved on the test sets. The cross-validated estimate of the prediction error, ${\widehat{\in }}_{cv}$, is then given as

6 $${\widehat{\in }}_{cv}=\frac{1}{n}\sum_{i=1}^{n}\mathcal{L}({y}_{i},{\widehat{f}}_{-k}({x}_{i}))$$

where ${\widehat{f}}_{-k}$ is the model trained on all but the ${k}$th test subset, ${\widehat{y}}_{i}={\widehat{f}}_{-k}({x}_{i})$ is the predicted value for the real class label, ${y}_{i}$, of case ${x}_{i}$, which is an element of the ${k}$th subset^38^.

#### Performance metrics

The performance of the developed system was evaluated using four metrics, i.e., accuracy, precision, recall, and F1-score, where T.P is true positive, T.N is true negative, F.P is false positive, and F.N is false negative. These metrics are shown in Eqs. (7) to (10). The overall accuracy of the system was computed by averaging the training and testing accuracies. The remaining 3 metrics were calculated using the obtained testing matrices. The training and testing time of the system was were also recorded.

7 $$Accuracy=\frac{T.P+T.N}{T.P+T.N+F.P+F.N}\times 100$$

8 $$Precision=\frac{T.P}{T.P+F.P}\times 100$$

9 $$Recall=\frac{T.P}{T.P+F.N}\times 100$$

10 $$F1-Score=2*\frac{Precision\times Recall}{Precision+Recall}\times 100$$

### Statistical analysis

An ANOVA with repeated measures was performed using IBM Statistical Package for Social Sciences (SPSS) Version 26.0, on a Windows 10 machine to investigate whether a statistically significant difference existed between the reported testing accuracies of various image dimensions and video resolutions for static and dynamic signs respectively. This was followed by post hoc analysis with a Bonferroni adjustment to conduct pairwise comparisons between the testing accuracies.

## Results and discussion

**Figure 7:**
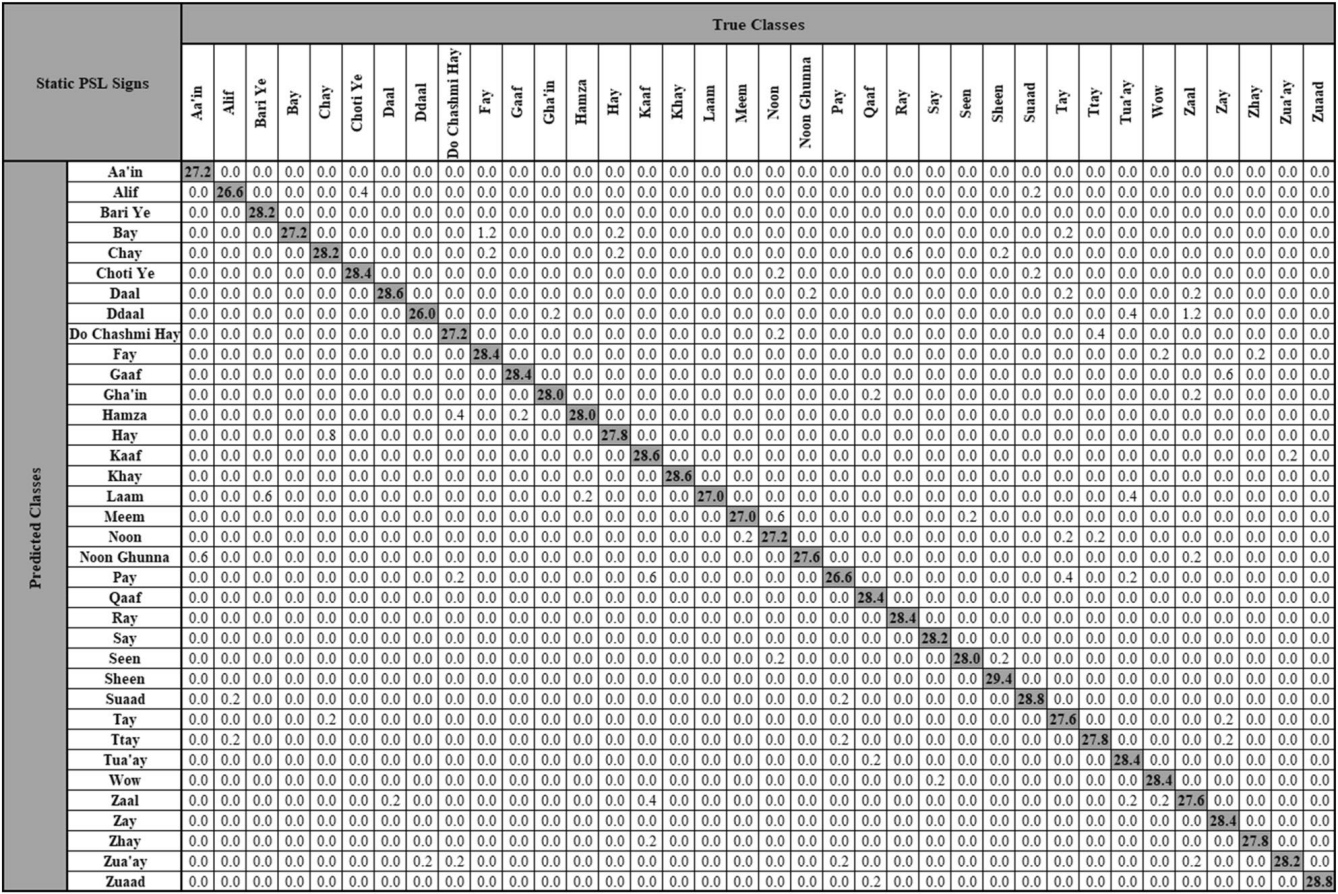
Confusion matrix of static PSL signs at 750 × 750 image dimensions and 500 bags.

The samples and details of the data collected per participant are mentioned in Table 2. In this study, fivefold cross validation was applied on the obtained codebook for static and dynamic signs, yielding five training codebooks and five testing codebooks for each K-cluster value of Bags used. As a size of 500 Bags was used for static signs with four different image scale sizes, as previously mentioned, a total of 20 models were trained for static images. The number of images used in each model were 4096 for training and 1024 for testing. The subsequent training and testing accuracies obtained from these 20 models are shown in Table 3 and their performance metrics in Table 4. The overall accuracies were obtained by averaging the training and testing accuracies of each model. The image scale size of 0.250 with 750 × 750 image dimensions and using 500 Bags yielded the highest overall classification accuracy for static signs of PSL alphabets, i.e., 97.80%. This 750 × 750 image dimensions also resulted in the highest precision, recall and F1-score that were computed using the testing matrices, as shown in Table 4. Figure 7 shows the confusion matrix of the testing model, which was obtained by averaging the testing confusion matrices of all the five models.

**Table 2:** PSL data per participant and total collected data.

Participant	Image samples total (min, max)	Video samples total (min, max)	Video duration in seconds Total (min, max)	Video frames Total (min, max)
1	511 (10, 16)	32 (10, 11)	80.26 (1.33, 3.68)	4755 (78, 216)
2	392 (10, 16)	38 (12, 14)	58.20 (0.71, 2.55)	3447 (42, 149)
3	423 (10, 15)	34 (10, 12)	69.16 (1.15, 3.57)	4138 (68, 215)
4	520 (11, 17)	35 (10, 14)	45.24 (0.70, 3.12)	2700 (40, 187)
5	514 (12, 16)	35 (10, 13)	66.31 (1.29, 3.07)	3915 (78, 185)
6	547 (15, 16)	40 (13, 14)	91.99 (1.08, 3.67)	5523 (65, 221)
7	548 (15, 16)	36 (11, 13)	91.82 (1.03, 5.09)	5532 (62, 307)
8	549 (13, 17)	34 (10, 13)	59.09 (0.92, 2.87)	3560 (55, 173)
9	547 (14, 17)	31 (10, 11)	61.71 (1.33, 3.22)	3717 (80, 194)
10	569 (15, 16)	38 (12, 13)	131.69 (1.96, 4.93)	7937 (118, 297)
Total	5120	353	755.47	45,224

**Table 3:** Classification accuracies for static signs at 500 bags.

Image dimensions (scale)	Model 1	Model 2	Model 3	Model 4	Model 5	Overall
**1500 × 1500 (0.500)**						
Training	94.80	94.90	95.10	95.60	95.10	95.10
Testing	95.21	96.19	96.09	96.00	96.68	96.03
Overall	95.01	95.55	95.60	95.80	95.89	95.57
**1225 × 1225 (0.375)**						
Training	96.60	96.60	96.10	96.40	96.10	96.36
Testing	97.66	97.07	97.66	96.39	98.05	97.37
Overall	97.13	96.84	96.88	96.40	97.08	96.86
**750 × 750 (0.250)**						
Training	97.40	97.30	97.80	97.40	97.40	97.46
Testing	98.24	98.05	97.95	98.73	97.75	98.14
Overall	97.82	97.68	97.88	98.07	97.58	**97.80**
**375 × 375 (0.125)**						
Training	96.00	95.80	96.00	95.80	95.90	95.90
Testing	96.48	96.88	96.29	96.39	96.39	96.49
Overall	96.24	96.34	96.15	96.10	96.15	96.19

Significant values are in bold.

**Table 4:** Performance metrics for static signs at 500 bags.

Image dimensions (scale)	Overall accuracy (%)	Precision (%)	Recall (%)	F1-Score (%)	Training time (s)	Testing time (s)
1500 × 1500 (0.500)	95.57	96.14	96.00	96.03	318.67	21.84
1225 × 1225 (0.375)	96.86	97.41	97.35	97.36	308.68	20.11
750 × 750 (0.250)	**97.80**	**98.17**	**98.14**	**98.14**	303.71	19.82
375 × 375 (0.125)	96.19	96.55	96.48	96.48	**297.15**	**19.49**

Significant values are in bold.

A repeated measures ANOVA with a Greenhouse–Geisser correction determined that mean testing accuracies for static signs differed statistically significantly between various image dimensions (F(2.027, 8.109) = 16.130, *p* < 0.001). Post hoc analysis with a Bonferroni adjustment revealed that there was a statistical significance between the testing accuracies of 750 × 750 and 1500 × 1500 dimensions (2.11 (95% CI 0.42 to 3.80), *p* < 0.023), and 750 × 750 and 375 × 375 dimensions (1.66 (95% CI 0.69 to 2.63), *p* < 0.007), but not between 750 × 750 and 1225 × 1225 dimensions (0.78 (95% CI − 1.37 to 2.93), *p* = 0.922), 1225 × 1225 and 1500 × 1500 dimensions (1.33 (95% CI − 0.35 to 3.01), *p* = 0.110), 1225 × 1225 and 375 × 375 dimensions (0.88 (95% CI − 0.73 to 2.49), *p* = 0.338), and 375 × 375 and 1500 × 1500 dimensions (0.42 (95% CI − 0.81 to 1.71), *p* = 0.937).

**Figure 8:**
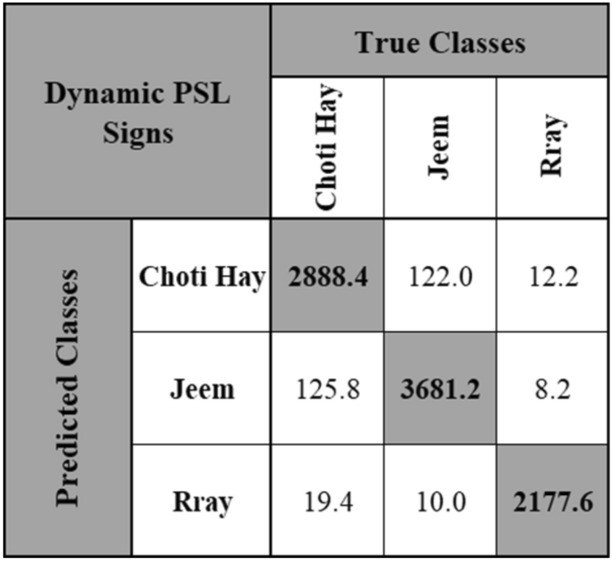
Confusion matrix of dynamic PSL signs at 480 × 270 video resolution and 200 bags.

Similarly, a size of 200 Bags was used for dynamic signs with three different video scale sizes, a total of 15 models were trained for dynamic signs. The number of video frames used for training in one model were 36,180 and 36,179 for the other four models and for testing in one model were 9044 and 9045 for the other four models. The subsequent training and testing classification accuracies obtained from these 15 models are shown in Table 5 and their performance metrics in Table 6. The video scale size of 0.250 with 480 × 270 video resolution and using 200 Bags yielded the highest overall classification accuracy for dynamic signs of PSL alphabets, i.e., 96.53%. This 480 × 270 video resolution also resulted in the highest precision, recall and F1-score, as shown in Table 6. Figure 8 shows its testing confusion matrix, which was obtained by averaging the testing confusion matrices of all the five models.

**Table 5:** Classification accuracies for dynamic signs at 200 bags.

Video resolution (scale)	Model 1	Model 2	Model 3	Model 4	Model 5	Overall
**720 × 405 (0.375)**						
Training	96.10	96.90	96.30	96.20	96.40	96.38
Testing	96.93	96.45	96.21	97.00	96.40	96.60
Overall	96.52	96.68	96.26	96.60	96.40	96.49
**480 × 270 (0.250)**						
Training	96.40	96.30	96.30	96.40	96.30	96.34
Testing	96.56	97.06	96.64	96.54	96.75	96.71
Overall	96.48	96.68	96.47	96.47	96.53	**96.53**
**240 × 135 (0.125)**						
Training	96.20	96.20	96.20	96.30	96.10	96.20
Testing	96.80	96.63	96.67	96.46	96.61	96.63
Overall	96.50	96.42	96.44	96.38	96.36	96.42

Significant values are in bold.

**Table 6:** Performance metrics for dynamic signs at 200 Bags.

Video resolution (scale)	Overall accuracy (%)	Precision (%)	Recall (%)	F1-score (%)	Training time (s)	Testing time (s)
720 × 405 (0.375)	96.49	96.85	96.79	96.82	273.13	6.05
480 × 270 (0.250)	**96.53**	**96.94**	**96.91**	**96.92**	267.13	5.89
240 × 135 (0.125)	96.42	96.88	96.85	96.87	**257.67**	**5.68**

Significant values are in bold.

A repeated measures ANOVA with a Greenhouse–Geisser correction determined that mean testing accuracies for dynamic signs did not differ statistically significantly between various video resolutions (F(1.343, 5.374) = 0.218, *p* = 0.727). Post hoc analysis with a Bonferroni adjustment further revealed that there was no statistical significance between the testing accuracies of 480 × 270 and 720 × 405 resolutions (0.11 (95% CI − 0.76 to 0.98), *p* = 1.000), 480 × 270 and 240 × 135 resolutions (0.08 (95% CI − 0.36 to 0.51), *p* = 1.000), and 240 × 135 and 720 × 405 resolutions (0.04 (95% CI − 0.64 to 0.72), *p* = 1.000).

For the collection of data, recruiting participants of different race, gender, age, height and skin colour, added variations to the collected dataset, such as different skin colours, hand size and so on. Asking the participants to perform the hand signs as they naturally would, caused variations in the orientation of the signs being performed, and minor variations due to different joint flexibility of the participants. By varying the height and distance between the camera and the participant according to the participants comfort also added variations in the scale of the data being collected. The data collected only required the hand to be captured. If the data of PSL sentences was captured, also collecting the facial expressions of the participants would increase the complexity of the system being developed.

The black background and clothing conditions helped in the thresholding technique used during segmentation, as the skin colour in grayscale was easily distinguished from the background and clothes. During the video segmentation, all the frames in the video had to be of the same size, in order save them for further processing. This issue was resolved by applying zero padding to the videos. This was done by finding the maximum dimensions from each video’s segmented frames and using that as a reference value to apply zero padding to the frames with lesser dimensions. This resulted in a uniform resolution size for that specific video. Zero padding was an effective technique for the dataset used in this study as the background chosen for the collected data was black and by applying zero padding black pixels were added to the videos as 0 represents black when the pixels of images are visualized.

The training and testing time obtained for the models decreased as the dimensions of the data was decreased. This suggests that as the number of pixels and thus the features decreased, the time required to train and test the models also decreased. However, this faster computation time did not result in higher classification accuracies.

Table 7 shows comparison between the studies performed on static SL and Table 8 compares studies performed on dynamic SL. the A similar study by, Dardas et al.^8^, used the Bag-of-features technique with SIFT and SVM to obtain 96.23% accuracy using 10 signs of static American SL with cluttered background. Another study by Farman Shah et al.^30^, used SURF with SVM but obtained 15.41% accuracy and the final reported accuracy using Histogram of Oriented Gradient (HOG) and SVM was 91.98%, which was also the highest classification accuracy reported, to the best of my knowledge, using static PSL alphabets. Our method yielded a 97.80% accuracy which exceeds the previous studies performed for static PSL alphabets. Studies by Abiyev et al.^10^ and Barbhuiya et al.^11^ used deep learning technique in combination with SVM and Cheng et al.^21^ used DTW mapping with SVM to obtain high classification accuracies. Joshi et al.^35^ used feature-level fusion techniques such as canonical correlation analysis (CCA) and discriminant correlation analysis (DCA) for their shape-based features to achieve high recognition accuracies.

**Table 7:** Comparison with other static SL methods.

Static method	Number of signs used (total image samples)	Accuracy (%)
Dardas et al.^8^	10 (1000)	96.23
Abiyev et al.^10^	24 (34,627)	99.90
Barbhuiya et al.^11^	36 (22,634)	99.82 and 99.76
Dahmani et al.^17^	30 (2880)	96.88
Cheng et al.^21^	39 (21,450)	99.03
Athira et al.^24^	24 (900)	90.10
Shah et al.^27^	37 (3414)	77.18
Ahmed et al.^28^	10 (600)	83.00
Shah et al.^30^	36 (6633)	91.98
Joshi et al.^35^	26 Indian SL (2300) and 10 American SL (418)	98.60 and 98.80
Our method	36 (5120)	97.80

**Table 8:** Comparison with other dynamic SL methods.

Dynamic method	Number of signs used (total video/frame samples)	Accuracy (%)
Singha et al.^5^	40 (11,600)	92.23
Tamiru et al.^12^	52 (1710 videos)	98.06
Charles et al.^19^	Not specified (20 videos with each over 40,000 frames)	75.00
Athira et al.^24^	2 (700 videos)	89.00
Cabrera et al.^25^	249 (2241 frames)	96.27
Our method	3 (353 videos and 45,224 frames)	96.53

Cabrera et al.^25^ used neural networks to detect skin colour and then extract features from their 2241 keyframes extracted from 249 videos. Tamiru et al.^12^ extracted 34 shape, motion and colour features to obtain their high classification accuracy. Shazia Saqib et al.^31^, used dynamic PSL words with CNN with Levenshtein distance to obtain 90.79% accuracy. No previously performed study has classified dynamic PSL alphabets, to the best of my knowledge, so the classification accuracy of 96.53% for dynamic PSL signs cannot be compared to any PSL study.

The limitations of this study were that the dataset collected used only uniform lighting and uniform background conditions and the data was only captured with the participant facing the camera, i.e., only from one angle using their dominant right hand. Furthermore, the system was developed in such a way that it used offline testing along with the offline training.

For future work, a PSL dataset could be created that uses various lighting and complex background conditions. The data of the signer could be captured from multiple angles. More participants can be recruited, to increase the size of the dataset. The system could also be implemented using real-time testing of the trained models. The developed system can be implemented in comparison other sign languages.

## Conclusion

The purpose of this study was to collect data of static and dynamic PSL alphabets and to develop a vision-based system for their recognition using BoW and SVM techniques. 36 static PSL alphabet signs and 3 dynamic PSL alphabet signs were collected with uniform background, uniform lighting at various orientations and scale, from 10 native signers of PSL and used as input in the developed system. The data was resized to various scales, segmented and converted into Bag-of-Words by finding the Euclidean distance between SURF descriptors and clustered value obtained by K-means clustering. The obtained codebooks were trained using SVM and tested to obtain the highest overall classification accuracy of 97.80%, precision of 98.17%, recall of 98.14% and F1-Score of 98.14% of for static PSL signs. For dynamic PSL signs an overall accuracy of 96.53%, precision of 96.94%, recall of 96.91% and F1-Score of 96.92% was obtained.
